# Biofilm associated genotypes of multiple antibiotic resistant *Pseudomonas aeruginosa*

**DOI:** 10.1186/s12864-021-07818-5

**Published:** 2021-07-26

**Authors:** James Redfern, Janine Wallace, Alex van Belkum, Magali Jaillard, Elliot Whittard, Roobinidevi Ragupathy, Joanna Verran, Peter Kelly, Mark Charles Enright

**Affiliations:** 1 Faculty of Science and Engineering, Manchester Metropolitan University, Chester Street, Manchester, United Kingdom; 2 bioMérieux SA, La Balme les Grottes, France

**Keywords:** *Pseudomonas aeruginosa*, Genomics, GWAS, Biofilm

## Abstract

**Background:**

*Pseudomonas aeruginosa* is a ubiquitous environmental microorganism and also a common cause of infection. Its ability to survive in many different environments and persistently colonize humans is linked to its presence in biofilms formed on indwelling device surfaces. Biofilm promotes adhesion to, and survival on surfaces, protects from desiccation and the actions of antibiotics and disinfectants.

**Results:**

We examined the genetic basis for biofilm production on polystyrene at room (22 °C) and body temperature (37 °C) within 280 *P. aeruginosa*. 193 isolates (69 %) produced more biofilm at 22 °C than at 37 °C. Using GWAS and pan-GWAS, we found a number of accessory genes significantly associated with greater biofilm production at 22 °C. Many of these are present on a 165 kb region containing genes for heavy metal resistance (arsenic, copper, mercury and cadmium), transcriptional regulators and methytransferases. We also discovered multiple core genome SNPs in the A-type flagellin gene and Type II secretion system gene *xpsD*. Analysis of biofilm production of isolates of the MDR ST111 and ST235 lineages on stainless-steel revealed several accessory genes associated with enhanced biofilm production. These include a putative translocase with homology to a *Helicobacter pylori* type IV secretion system protein, a TA system II toxin gene and the alginate biosynthesis gene *algA*, several transcriptional regulators and methytransferases as well as core SNPs in genes involved in quorum sensing and protein translocation.

**Conclusions:**

Using genetic association approaches we discovered a number of accessory genes and core-genome SNPs that were associated with enhanced early biofilm formation at 22 °C compared to 37 °C. These included a 165 kb genomic island containing multiple heavy metal resistance genes, transcriptional regulators and methyltransferases. We hypothesize that this genomic island may be associated with overall genotypes that are environmentally adapted to survive at lower temperatures. Further work to examine their importance in, for example gene-knockout studies, are required to confirm their relevance. GWAS and pan-GWAS approaches have great potential as a first step in examining the genetic basis of novel bacterial phenotypes.

**Supplementary Information:**

The online version contains supplementary material available at 10.1186/s12864-021-07818-5.

## Background

*Pseudomonas aeruginosa* is a mono-flagellate, Gram-negative bacterium that is present in most environments. A frequent coloniser of humans, other animals and plants, *P. aeruginosa* is also a very common opportunistic pathogen able to grow at a variety of temperatures [1]. The species is a leading cause of severe, life-threatening nosocomial human infections and the major pathogen associated with lung infections of patients with cystic fibrosis. This microorganism has been classified as one of the major species associated with multiple antimicrobial resistance of urgent public health concern by the Infectious Diseases Society of America [2], Centers for Disease Control and Prevention [3] and the World Health Organization [4].

A variety of typing methods including multilocus sequence typing (MLST) [5] and more recently, genomic sequencing studies [6–9] have shown that *P. aeruginosa* has a non-clonal, epidemic population structure in which successful clones occasionally arise and are globally transmitted [10]. Multidrug resistant (MDR) isolates of lineages associated with outbreaks of infection belonging to MLST Sequence Types (STs) 111 and ST235 are two such ‘high risk’ clones that have global distributions and numerous and transferable antibiotic resistances [11]. Isolates of these lineages contain a large number of horizontally transferred β-lactamase genes [8] and are highly virulent in comparison to a third, less significant lineage - ST175 [12].

*P. aeruginosa* has many phenotypic features that promote its success as a pathogen. In common with other bacterial pathogens *P. aeruginosa* usually exists within biofilms. These are complex microbial communities associated with extracellular polymeric matrices that help bacteria resist desiccation, mechanical removal and the actions of antibiotics and disinfectants [13]. In human tissue, biofilm-associated *P. aeruginosa* are difficult to eradicate and represent infectious foci that can lead to serious systemic disease. *P. aeruginosa* biofilms are found at surface-liquid, surface-air and liquid-air interfaces, and are a significant clinical problem in wounds and the cystic fibrosis lung [14]. Polymicrobial biofilms are also recognized as important niches for MDR evolution, as they represent a significant reservoir for horizontal gene transfer within and between bacterial species [15].

In addition to comprehending *in vivo* biofilm formation, understanding *P. aeruginosa* biofilm development and persistence on other surfaces is required. Stainless-steel is the most common surface material used in many industries where control of microorganisms is important, including healthcare. Properties of stainless-steel include resistance to corrosion, easiness to clean and a level of hardness likely to limit scratches and other defects [16]. However, many of these surfaces have been shown to allow the formation of biofilms, for example in hard-to-clean locations, and those with a favourable environment, such as sinks and pipes [17]. ST111 isolates have been shown to produce significantly more biofilm compared to those from non-high-risk clones [18] whilst high-risk clones are over-represented in locations such as hospital sink pipes [19]. A recent study strongly implicates the presence of *P. aeruginosa* biofilm in hospital waste-water pipes as being responsible for an outbreak of infection due to MDR ST111 and ST235 in a haematology unit [20] and policies promoting the replacement of sink units to reduce such sources of infection have been introduced in the United Kingdom [21].

There is a need to better understand the clinical importance of biofilms in hospital environments starting with how biofilm production changes when *P. aeruginosa* cells move from the hospital environment to the human body. Pathogen genomics is becoming a key tool for epidemiological surveillance of many species and can be used to guide clinical treatment and outbreak control [22, 23]. The large number of pathogen genomes and associated metadata in the public domain represents a rich and growing resource for bioinformatic investigations of pathogenicity, antibiotic resistance, vaccine susceptibility and other important phenotypes. Genome Wide Association Studies (GWAS) have been used to great effect in identifying genetic determinants contributing to disease pathology in human medicine. Thus far, microbial GWAS and more generic Whole Genome Sequencing (WGS) studies have focused on the molecular epidemiology of infectious disease and particularly on associations between the genome and antimicrobial resistance and / or pathogenicity. There is huge potential for such methods to help understand the genetic basis of other phenotypes, such as key processes that are important in microbial ecology or industrial microbiology [24, 25].

In this study we sought to find genes and core genome SNPs associated with biofilm production by examining associations within the *P. aeruginosa* pan-genome and biofilm-producing phenotypes in two experiments using a collection of 280 *P. aeruginosa* isolates whose genomes were sequenced in a previous study [6]. In the first we measured the density of biofilm produced by *P. aeruginosa* grown in polystyrene microtiter plates at ambient (22 °C) and body (37 °C) temperatures. In the second we measured the biofilm density of isolates from this larger collection belonging to the international MDR ST111 and ST235 lineages grown on stainless-steel.

## Results

### Temperature-dependent biofilm analysis

**Fig. 1:**
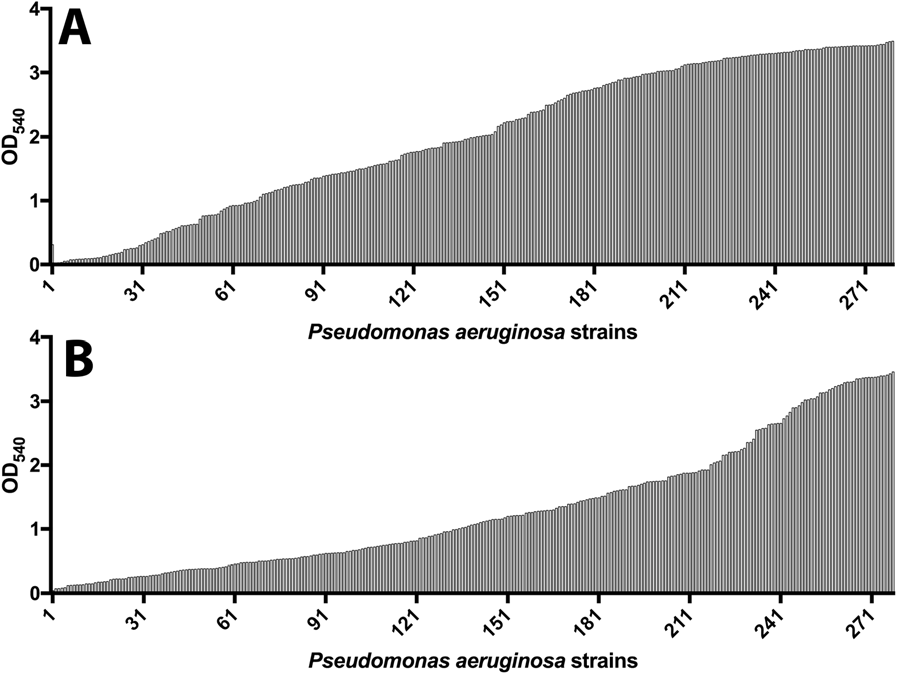
Optical density related to biofilm stained with 0.1 % crystal violet and solubilised in 30 % acetic acid at either 22 °C (A) or 37 °C (B) for 280 *Pseudomonas aeruginosa* isolates. *n* = 4 for each isolate/temperature condition

**Fig. 2:**
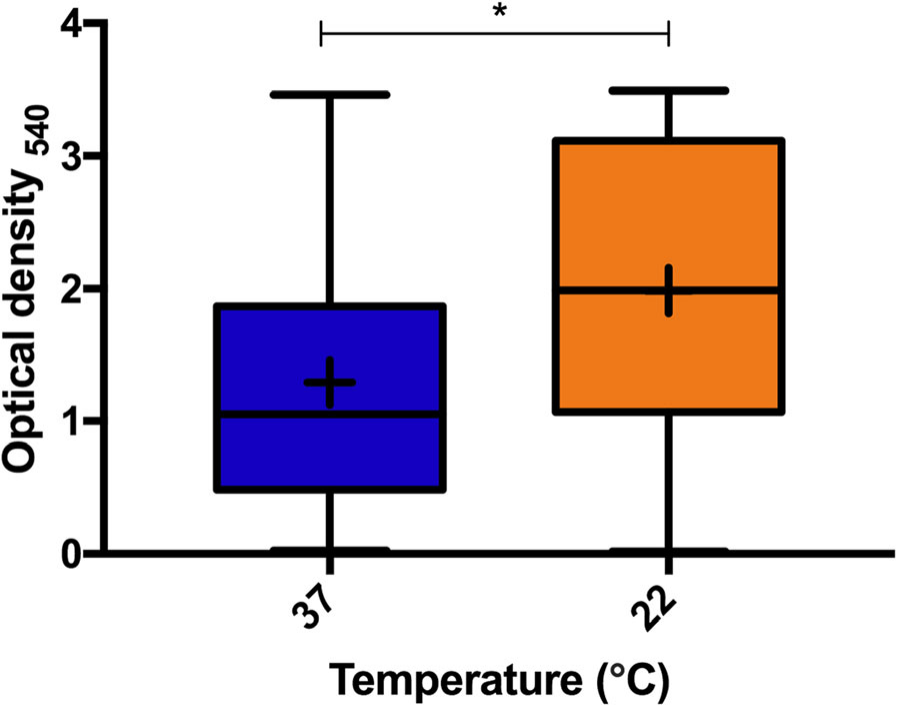
Min and max box plot demonstrating the variation in biofilm formation for 280 *Pseudomonas aeruginosa* isolates at two different temperatures as measured by optical density. Average values are denoted by +. Significance (*P* < 0.05), as assessed by T-Test is denoted by *. Each box plot represents data for 280 isolates

**Fig. 3:**
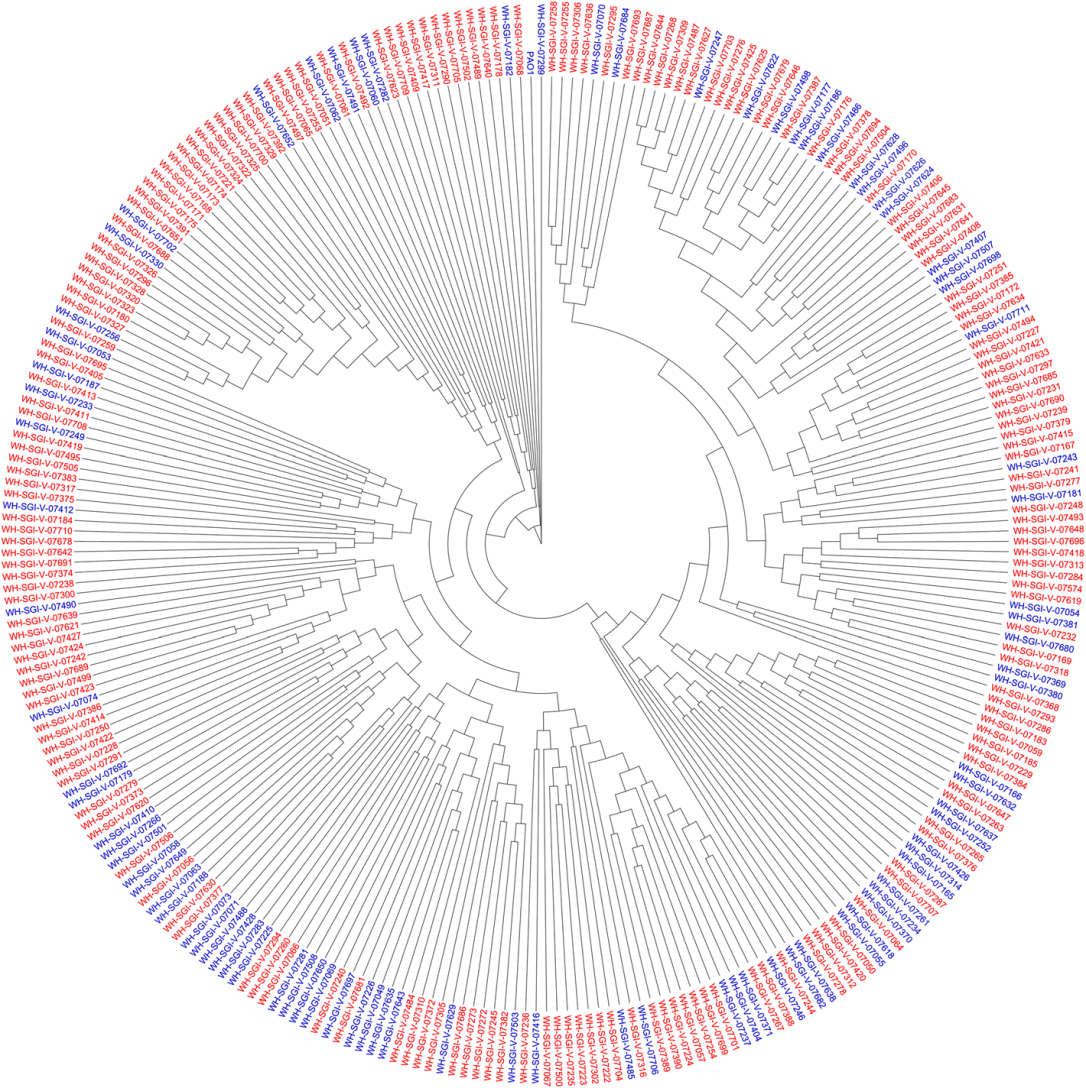
Unrooted maximum parsimony (MP) tree based on core genome SNPs of 280 *Pseudomonas aeruginosa* isolates. Tree is a consensus of 100 MP trees generated using kSNP3 [26]. Names highlighted red represent an isolate that produced more biofilm at 22 °C and names highlighted blue represent an isolate that produced more biofilm at 37 °C

Biofilm formation varied greatly between isolates at both 22 and 37 °C (Fig. 1). Of the 280 isolates studied, 69 % (*n* = 193) produced more biofilm at 22 °C (mean OD_540_ of 1.98) compared to 37 °C (mean OD_540_ measurement of 1.29). Isolates that produced more biofilm at 22 °C than at 37 °C had a mean OD_540_ measure of 2.35 at 22 °C and 0.97 at 37 °C. Isolates producing more biofilm at 37 °C had a mean OD_540_ measure of 1.16 at 22 °C and 2.02 at 37 °C. Isolates producing more biofilm at 22 °C belonged to 84 different MLST STs compared to 61 of isolates producing more biofilm at 37 °C. Isolates demonstrated more variation in OD540 measures when grown at 37 °C (mean = 1.291, standard deviation = 0.98, coefficient of variation = 76.12 %), than at 22 °C (mean of 1.983, standard deviation of 1.12, coefficient of variation = 56.34 %). (Fig. 2). Increased biofilm growth phenotype at 37 °C was widely distributed across the phylogeny as shown in Fig. 3.

Scoary analyses yielded 2,834 clusters of orthologous groups (COGs) and genes associated with the phenotypic trait of producing more biofilm at 22 °C compared to 37 °C (naïve *p* < 0.05) (Suppl. Table 3). None of these hits were significant using a Bonferroni corrected *p*-value of < 0.05 but 74 were significantly associated with this phenotype using the conservative, but less stringent, Benjamini-Hochberg correction at *p* < 0.05. Pyseer analysis using the same input gave 1,015 significant hits (naïve *p* < 0.05) and 155 using a lrt (likelihood - ratio test) *p* < 0.01 (Suppl. Table 4). The top 20 most significant hits using the two tests are shown in Table 1 with the 14 genes present in both lists shown in bold type. Scoary hits were ordered by Benjamini-Hochberg – adjusted *p*-values and Pyseer hits by lrt- *p*-values. All hits are shown in Suppl. Tables 3 and 4. 20/20 of the most significant hits identified by Scoary are present in the genome of isolate WH-SGI-V-07050 as are 15 / 20 using Pyseer and genes in Table 1 were therefore numbered according to this genome (when present). These genes include *acr3*, *arsC*, *arsR* and *arsH* - four genes involved in arsenic resistance / reduction as well as two methyltransferases and an integrating conjugative element (ICE) protein and an ICE relaxase. The gene *arsR*-family transcriptional regulators are considered to be important in many physiological processes, including biofilm production [27]. Presence of arsenic in bacterial cells has been demonstrated to affect biofilm synthesis as well as chemotaxis and motility [28], and there is a suggestion that arsenic can prevent the switch between planktonic and sessile lifestyle [29]. The presence of the flagellar protein FliC is also significantly associated with increased biofilm growth at 22 °C compared to 37 °C Pyseer (but not Scoary). Flagellar motility is well-known as a requirement for biofilm production in *P. aeruginosa*[30] and suppression of *fliC* has been linked to increasing temperature in *P. syringae*[31], with similar temperature-association of flagellar being reported in *Listeria monocytogenes* and *Proteus vulgaris*[32, 33]. The gene *clsA*, a cardiolipin synthase has demonstrated impact on biofilm formation in *E. coli *[34] although association with temperature has not been evaluated. The two methyltransferases and in particular the SAM (S-adenosylmethionine) – dependent methyltransferase may have a role in N-acyl-homoserine lactone synthesis, the key molecules in *P. aeruginosa* quorum sensing (QS) [35]. QS has long been known to play a central role in biofilm formation in *P. aeruginosa *[36] however further work would be required to establish the importance of these methyltransferases in QS and biofilm production.

**Table 1:** Ranked list of the most significant genes / COGs associated with greater biofilm growth at 22^∘^C than 37^∘^C using Scoary and Pyseer

**SCOARY**						
	COG / Gene	Function	Presence	OR	Ben p	Example (isolate_gene position)
**1**	**group_8321**	**Methyltransferase**	**68**	**6.389**	**0.018**	**WH-SGI-V-07050_00375**
**2**	**group_15072**	**Hypothetical**	**80**	**5.044**	**0.018**	**WH-SGI-V-07050_00461**
**3**	**group_1519**	**SAM-dependent methyltransferase**	**79**	**4.932**	**0.018**	**WH-SGI-V-07050_00374**
**4**	**group_15041**	**Integrating conjugative element protein**	**75**	**5.251**	**0.018**	**WH-SGI-V-07050_00387**
**5**	**group_11043 (arsC_1)**	**Arsenate reductase (glutaredoxin)**	**78**	**4.823**	**0.018**	**WH-SGI-V-07050_00379**
**6**	**acr3**	**ACR3 family arsenite efflux transporter**	**78**	**4.823**	**0.018**	**WH-SGI-V-07050_00380**
**7**	**arsC_2**	**Arsenate reductase**	**78**	**4.823**	**0.018**	**WH-SGI-V-07050_00381**
**8**	**group_11046**	**ArsR family transcriptional regulator**	**78**	**4.823**	**0.018**	**WH-SGI-V-07050_00382**
**9**	**group_2314**	**Hypothetical**	**71**	**4.823**	**0.018**	**WH-SGI-V-07050_00373**
**10**	**arsH_1**	**Arsenical resistance protein**	**78**	**4.823**	**0.018**	**WH-SGI-V-07050_00378**
**11**	**ydfG**	**NADP-dependent 3-hydroxy acid dehydrogenase**	**73**	**5.015**	**0.018**	**WH-SGI-V-07050_00440**
**12**	**group_15062**	**TetR/AcrR family transcriptional regulator**	**73**	**5.015**	**0.018**	**WH-SGI-V-07050_00441**
**13**	**group_11053**	**Integrating conjugative element relaxase, PFGI-1 class**	**77**	**4.715**	**0.018**	**WH-SGI-V-07050_00417**
14	qorB	Quinone oxidoreductase	77	4.715	0.018	WH-SGI-V-07050_00419
**15**	**group_832**	**Hypothetical**	**77**	**4.715**	**0.018**	**WH-SGI-V-07050_00384**
16	group_11042	Hypothetical	63	5.658	0.018	WH-SGI-V-07050_00376
17	group_15038	Integrating conjugative element protein pill, pfgi-1	76	4.608	0.018	WH-SGI-V-07050_00383
18	group_15039	TIGR03759 family integrating conjugative element protein	76	4.608	0.018	WH-SGI-V-07050_00385
19	group_15040	Transglycosylase SLT domain-containing protein	76	4.608	0.018	WH-SGI-V-07050_00386
20	group_11058	MgtC/SapB family protein	84	4.203	0.018	WH-SGI-V-07050_00439
**PYSEER**						
	COG / Gene	Function	Presence	Beta	lrt p	Example
**1**	**group_8321**	**Methyltransferase**	**68**	**1.9200**	**0.0001**	**WH-SGI-V-07050_00375**
**2**	**group_15072**	**Hypothetical**	**80**	**1.6800**	**0.0001**	**WH-SGI-V-07050_00461**
**3**	**group_15041**	**Integrating conjugative element protein**	**75**	**1.6500**	**0.0002**	**WH-SGI-V-07050_00387**
4	group_11114	Hypothetical	140	1.5000	0.0002	WH-SGI-V-07050_02801
**5**	**group_1519**	**SAM-dependent methyltransferase**	**79**	**1.5700**	**0.0003**	**WH-SGI-V-07050_00374**
6	group_3009	4-alpha-glucanotransferase	72	1.3700	0.0003	WH-SGI-V-07051_05729
**7**	clsA	Major cardiolipin synthase ClsA	42	1.6900	0.0003	WH-SGI-V-07066_05171
**8**	**ydfG**	**NADP-dependent 3-hydroxy acid dehydrogenase**	**73**	**1.6200**	**0.0003**	**WH-SGI-V-07050_00440**
**9**	**group_15062**	**TetR/AcrR family transcriptional regulator**	**73**	**1.6200**	**0.0003**	**WH-SGI-V-07050_00441**
**10**	**group_11043 (arsC_1)**	**Arsenate reductase (glutaredoxin)**	**78**	**1.5500**	**0.0003**	**WH-SGI-V-07050_00379**
**11**	**acr3**	**ACR3 family arsenite efflux transporter**	**78**	**1.5500**	**0.0003**	**WH-SGI-V-07050_00380**
**12**	**arsC_2**	**Arsenate reductase**	**78**	**1.5500**	**0.0003**	**WH-SGI-V-07050_00381**
**13**	**group_11046**	**ArsR family transcriptional regulator**	**78**	**1.5500**	**0.0003**	**WH-SGI-V-07050_00382**
**14**	**arsH_1**	**Arsenical resistance protein**	**78**	**1.5500**	**0.0003**	**WH-SGI-V-07050_00378**
15	fliC	Flagellin	109	1.2400	0.0004	WH-SGI-V-07064_05796
16	group_5435	Alpha/beta fold hydrolase	101	-1.1300	0.0004	WH-SGI-V-07049_05847
17	group_5843	Hypothetical	35	1.8200	0.0004	WH-SGI-V-07066_05167
**18**	**group_2314**	**Hypothetical**	**71**	**1.5400**	**0.0004**	**WH-SGI-V-07050_00373**
**19**	**group_832**	**Hypothetical**	**77**	**1.5100**	**0.0004**	**WH-SGI-V-07050_00384**
**20**	**group_11053**	**Integrating conjugative element relaxase, PFGI-1 class**	**77**	**1.5000**	**0.0005**	**WH-SGI-V-07050_00417**

Presence = number of isolates (of 280) gene is present in. OR = Odds ratio and Ben p = Benjamini-Hochberg corrected *p* value for Scoary

Beta = slope of regression and lrt p = likelihood ratio test *p* value using Pyseer. Genes in bold are present in both lists

**Fig. 4:**
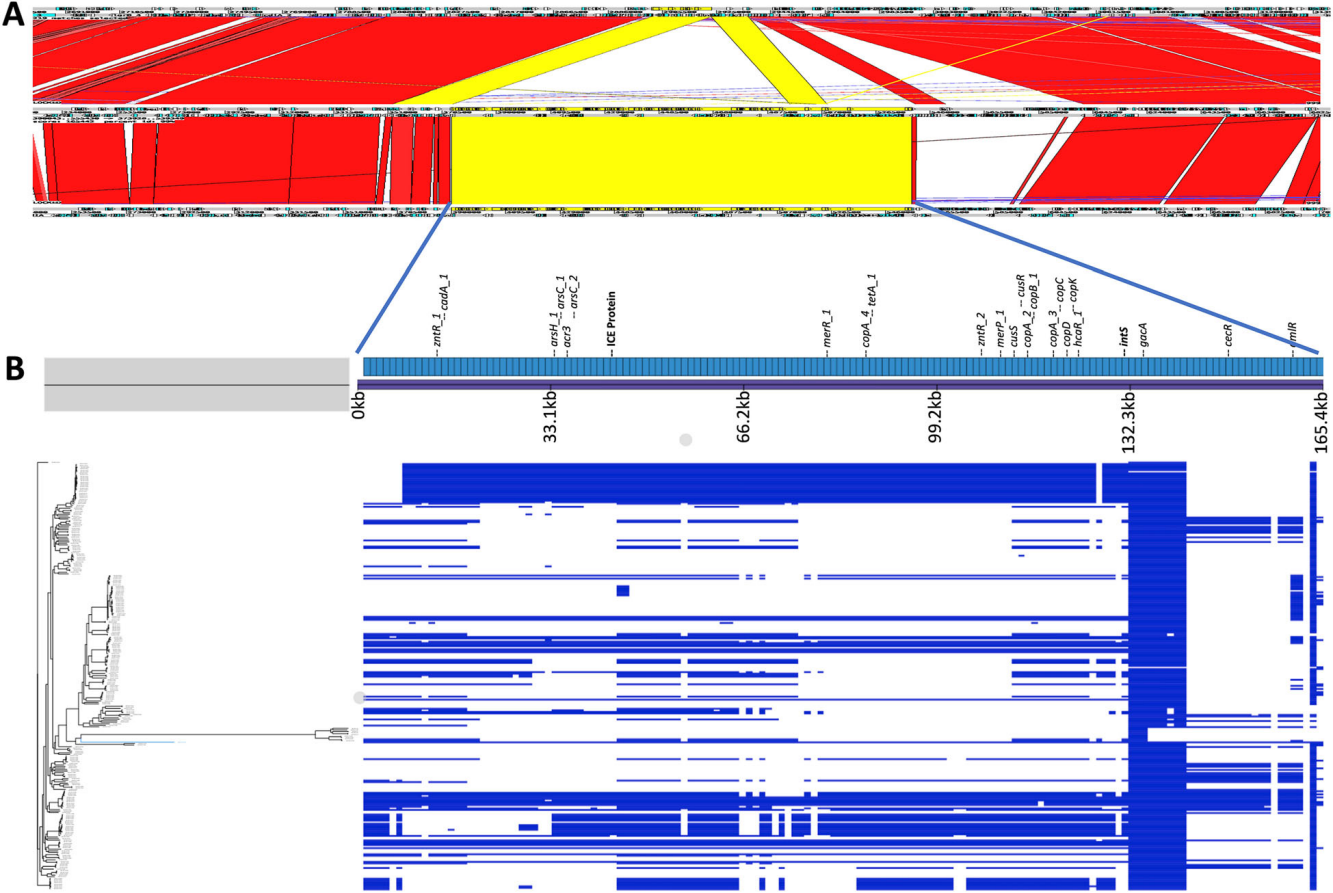
Analysis of a 165,376 bp accessory region of the *P. aeruginosa* genome present in isolate WH-SGI-V-07050. Panel **A** shows a genomic comparison of a region shared between reference genome FDAARGOS-532 (GenBank accession GI:1,519,006,927) [middle] and isolate WH-SGI-V-07050 [bottom] showing its absence in the genome of PAO1 [top]. Artemis Comparison Tool was used to visualise pairwise blastp results. Panel **B** shows the presence or absence of genes in this region in all study isolates visualised using Phandango

The majority (20/26) of the most significant hits presented in Table 1 are in close proximity to each other in the WH-SGI-V-07050 chromosome – genes numbered 373 (WH-SGI-V-07050_00373 in Table 1) to 461 (WH-SGI-V-07050_00461). These genes are present in between 63 and 80 of the 280 study isolates. Comparative analysis of DNA in this region from the genome of isolate WH-SGI-V-07050 with other study isolate genomes show that this region is 165,376 bp long and contains 149 genes starting from WH-SGI-V-07050_349 to WH-SGI-V-07050_498. A Blastn search of this region showed > 99 % DNA similarity over the entire region with several finished genomic sequences including strain FDAARGOS-532 (GenBank accession GI:1519006927). A manual alignment of this genome with those of WH-SGI-V-07050 and PAO1 (GI:110645304) was performed using Artemis Comparison Tool [37] (Fig. 4). This shows that this region is not present in strain PAO1 except for a region of 13,273 bp corresponding to bases 2,923,150 to 2,936,423 of PAO1 common to all three genomes. This conserved region contains ten open reading frames including *pgsA* at the 5’ end and the hypothetical protein gene PAO1_02672 at the 3’ end.

We used Phandango to visualise the genomes of the 280 isolates in this study for the presence (or absence) of genes corresponding to WH-SGI-V-07050_349 to WH-SGI-V-07050_498. Figure 4 shows that most of these genes are present in the genomes of many isolates and that their distribution is not strongly associated with core genome phylogeny although only a small number of genomes share the total length of this region with WH-SGI-V-07050 (shown in blue in Fig. 4). Besides containing a cluster of arsenic resistance genes (see above) this region also contains genes encoding heavy metal-resistance genes for copper (*copA2, copA_3, copA_4, copB_1, copC, copD, copK*), mercury (*merR_1, merP_1*), and cadmium (*cadA_1*) that (with the exception of *cadA*) are significantly associated with the phenotype of producing more biofilm at ambient compared to body temperature (Scoary and Pyseer ranked hits are shown in Table 1 (and raw data in Suppl. Tables 3 and 4). The region also contains a gene for tetracycline resistance (*tetA_1*) that is significantly associated with this phenotype as well as several transcriptional regulators: - *zntR_1*, *zntR_2*, *cusR* (adjacent to sensor kinase gene *cusS*), *hcaR_1*, *cecR* and *dmlR_24*. Also present in this region are a cluster of nine genes that are present in between 260 and 278 of the 280 genomes studied. These genes include the response regulator gene *gacA*, a key gene regulating transition from a planktonic to biofilm lifestyle [38], a putative transcriptional regulator gene as well as a colicin receptor gene. The architecture of this region suggests a history of recent recombination events and this is supported by the presence in this region of an Integrative and Conjugative Element (ICE) protein and a prophage integrase that would strongly suggest that these gene clusters have been mobilised by conjugative transposition and phage transduction events into independent lineages.

Transposon mutagenesis can be used to help elucidate the relative importance of individual candidate genes to observed biofilm phenotypes. The *arsR* family transcriptional regulator (Table 1) is present in 78 of 280 isolates and is not present in PAO1. It is distinct from the *arsR* gene present in 275 / 280 of our isolates that forms an arsenic resistance operon with *arsB* and *arsC* in PAO1. We found that two separate transposon mutants of the *arsR* gene in PAO1 showed enhanced biofilm growth at 22 °C compared to 37 °C (Suppl. Figure 2). This is supportive of a role for this gene in biofilm production at ambient temperature although several future mutagenesis experiments would be required to support a key role for such genes in this temperature associated phenotype.

### Temperature associated core genome SNPs

**Fig. 5:**
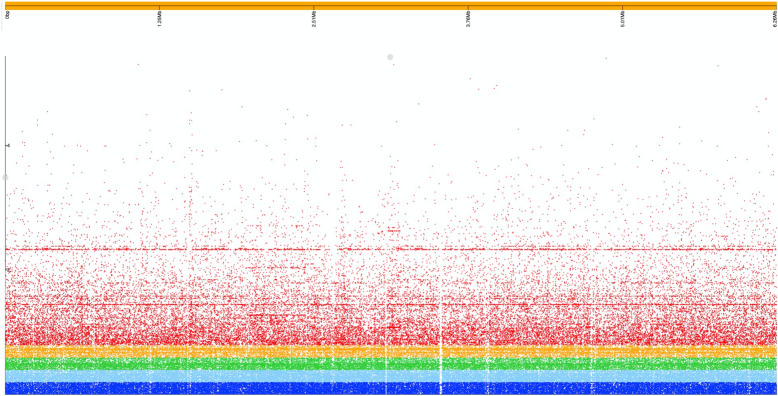
Manhattan plot showing core genome significance (lrt *p*-value) of SNPs in the PAO1 genome associated with moregreater biofilm production at 22 °C than at 37 °C. The Y-axis corresponds to lrt *p*- values. 37 °C and their position in the PAO1 chromosome

Pyseer analysis yielded 2081 significant SNPs (lrt *p* < 0.001) associated with increased biofilm production at 22 °C compared to 37 °C (Suppl. Table 5). All SNPs ranked by significance (lrt *p-*values on y-axis) are shown in a Manhattan Plot in Fig. 5 with their location in the PAO1 genome. The 30 most significant hits (ordered by lrt-adjusted *p*-values) relative to the PAO1 genome are shown in Suppl. Table 6. These include three genes with more than one SNP – Type II secretion system protein D, a sulfotransferase family protein and a nucleoside binding protein. Type II secretion systems are associated with pathogenesis and environmental survival in a number of species and involve the export of proteins to the extracellular biofilm matrix [39]. The possible role of sulfotransferases and the second most significant SNP - in *cysW*, a sulfate transport system permease gene, in biofilm production are unclear as is the role of PAO1 gene 00240, a nucleoside binding protein. The most significant SNP associated with increased biofilm production at 22 °C is in the PAO1 gene 04493, that codes for 1-acyl-sn-glycerol-3-phosphate acyltransferase. This enzyme and *quiP*, an acyl-homoserine lactone acylase gene, may be involved in interactions with QS systems as the major QS signalling molecules in *P. aeruginosa* are acyl-homoserine lactones. Other SNPs in genes that have previously been found to be involved in biofilm production or regulation are in *algX* and *algA*, genes that form part of the operon involved in the production of the well-characterized extracellular polysaccharide biofilm component alginate [40] that is associated with the hyper-mucosity phenotype observed in isolates from some cystic fibrosis patients. The response regulator *pleD* is known to play a role in motility and transition between sessile and motile forms in *Caulobacter crescentus*[41] and its homologue in PAO1, *dgcH*[42] is required for biofilm production. Less clear is the role of the helix-turn-helix transcriptional regulator *syrM.* Other significant SNPs in genes involved in cellular motility in Table 2 are the flagellar glycosyl transferase gene *fgtA* and a putative major fimbrial subunit gene *lpfA* that may facilitate bacterial attachment and promote biofilm formation [43]. A full list of SNPs associated with greater biofilm production at 22 °C than at 37 °C is shown in Suppl. Table 6.

**Table 2:** Details of genes / COGs associated with biofilm phenoptype on stainless-steel identified by Pyseer

**ST111**					
**Cog / Gene**	**Function**		**Presence**	**lrt-*p*value**	**Beta**
group_257	Type IV secretion system apparatus / Translocase	WH-SGI-V-07174_06314	16	0.005	-0.773
cusS_2	Sensor kinase CusS	WH-SGI-V-07168_02759	22	0.008	1.050
group_2942	HD-GYP domain-containing protein	WH-SGI-V-07174_02964	2	0.008	-1.820
group_2943	Hypothetical	WH-SGI-V-07174_02965	2	0.008	-1.820
group_2944	Hypothetical	WH-SGI-V-07174_02966	2	0.008	-1.820
group_2945	Transposase	WH-SGI-V-07174_02967	2	0.008	-1.820
group_2946	XRE family transcriptional regulator	WH-SGI-V-07174_02968	2	0.008	-1.820
group_2947	type II toxin-antitoxin system RelE/ParE family toxin	WH-SGI-V-07174_02969	2	0.008	-1.820
hin_4	DNA-invertase	WH-SGI-V-07174_02970	2	0.008	-1.820
group_2949	Tn3 family transposase	WH-SGI-V-07174_02971	2	0.008	-1.820
**ST235**					
**Cog / Gene**	**Function**		**Presence**	**lrt-*p*value**	**Beta**
group_2587	Type IV secretion system apparatus / Translocase	WH-SGI-V-07406_05831	5	0.000	1.640
group_2588	Hypothetical	WH-SGI-V-07406_05832	5	0.000	1.640
group_2593	HK97 gp10 family phage protein	WH-SGI-V-07406_05844	5	0.000	1.640
group_977	Methyltransferase domain protein	WH-SGI-V-07406_05845	5	0.000	1.640
group_129	Glycoside hydrolase family 19 protein	WH-SGI-V-07425_03679	4	0.001	1.920
group_2655	Hypothetical	WH-SGI-V-07425_03618	4	0.001	1.920
merP_2	Mercuric transport protein periplasmic component	WH-SGI-V-07176_04878	6	0.001	-2.240
group_953	Mercury resistance co-regulator MerD	WH-SGI-V-07176_04876	6	0.001	-2.240
group_596	Phage gp6-like head-tail connector protein	WH-SGI-V-07406_05848	12	0.001	1.560
dcm	DNA-cytosine methyltransferase	WH-SGI-V-07170_03311	19	0.002	2.600
algA_3	Alginate biosynthesis protein AlgA	WH-SGI-V-07170_03301	19	0.002	2.600
group_199	SAM-dependent methyltransferase, partial	WH-SGI-V-07176_05804	4	0.003	2.420
group_2651	Hypothetical	WH-SGI-V-07425_03614	2	0.005	1.510
group_2634	Hypothetical	WH-SGI-V-07425_01793	4	0.006	1.850
group_2635	Hypothetical	WH-SGI-V-07425_01794	4	0.006	1.850
group_2636	DUF3577 domain-containing protein	WH-SGI-V-07425_01796	4	0.006	1.850
group_2638	Hypothetical	WH-SGI-V-07425_01801	4	0.006	1.850
group_2639	TIGR03761 family integrating conjugative element protein	WH-SGI-V-07425_01809	4	0.006	1.850
frmR	Transcriptional repressor FrmR	WH-SGI-V-07425_04778	4	0.006	1.850
group_2677	Hypothetical	WH-SGI-V-07425_04812	4	0.006	1.850
group_994	Hypothetical	WH-SGI-V-07425_01799	4	0.006	1.850
group_1012	Uridylate kinase	WH-SGI-V-07496_01971	3	0.007	-3.470
group_1013	DUF3275 family protein	WH-SGI-V-07496_01976	3	0.007	-3.470
group_1014	SAM-dependent methyltransferase	WH-SGI-V-07496_01979	3	0.007	-3.470
group_1015	Hypothetical	WH-SGI-V-07496_01983	3	0.007	-3.470
group_1016	TIGR03759 family integrating conjugative element protein	WH-SGI-V-07496_01984	3	0.007	-3.470
group_1017	Type IV conjugative transfer system coupling protein TraD	WH-SGI-V-07496_01987	3	0.007	-3.470
group_1018	TIGR03747 family integrating conjugative element membrane protein	WH-SGI-V-07496_01988	3	0.007	-3.470
group_1019	Restriction endonuclease	WH-SGI-V-07496_01991	3	0.007	-3.470
group_1020	GIY-YIG nuclease family protein	WH-SGI-V-07496_01992	3	0.007	-3.470
group_1021	TIGR03745 family integrating conjugative element membrane protein	WH-SGI-V-07496_01998	3	0.007	-3.470
group_1022	TIGR03746 family integrating conjugative element protein	WH-SGI-V-07496_02000	3	0.007	-3.470
group_1023	TIGR03752 family integrating conjugative element protein	WH-SGI-V-07496_02002	3	0.007	-3.470
group_1024	Conjugative transfer ATPase	WH-SGI-V-07496_02004	3	0.007	-3.470
group_1025	TraI domain-containing protein	WH-SGI-V-07496_02019	3	0.007	-3.470
ftsH4	ATP-dependent zinc metalloprotease FtsH 4	WH-SGI-V-07496_03490	3	0.007	-3.470

Presence = number of genomes gene is present in /25 for ST111 and /23 for ST235

### Biofilms on stainless-steel

Of the isolates assessed for biofilm production on stainless-steel (Suppl. Figures 1), 40 % (*n* = 10 out of 25) ST111 and 52 % (*n* = 13 out of 23) ST235 were considered high density biofilm producers. This demonstrates a large intra-clone variation of the biofilm phenotype. However, the variation observed in one clone was similar to the other (i.e. not significantly different), suggesting the intra-clone variation may be a common feature of other clones.

**Fig. 6:**
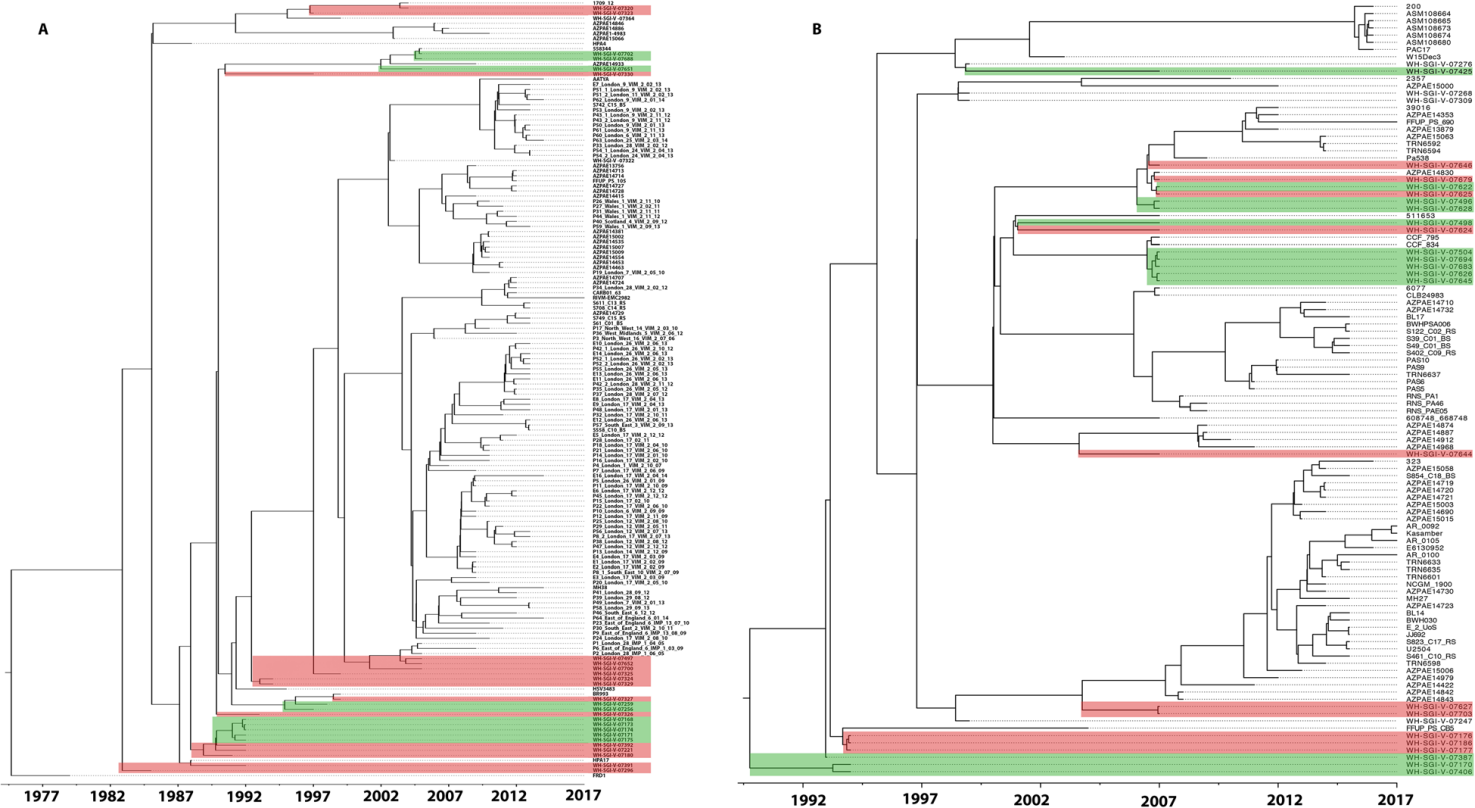
Bayesian evolutionary analysis sampling tree representative of the ST111 (**A**) and ST235 (**B**) strains included within analysis. Horizontal distance is indicative of time in years. Isolates included in the biofilm on stainless-steel phenotype assay (Suppl. Figure 1) are overlaid with their designated phenotype – high density biofilm producers (green) and low-density biofilm producers (red)

BEAST 2 analysis suggests that this sample of 153 ST111 isolates diverged from a common ancestor ≅ 43 years ago (Fig. 6.a), with a pan-genome of 15,488 genes. The 107 ST235 isolates included in this study had a pan-genome of 15,178 genes and diverged from a common ancestor ≅ 28 years ago (Fig. 6.b), suggesting that the two lineages emerged within approximately 15 years of each other. When considered alongside biofilm phenotype, those isolates sharing phylogenetic similarity display similar biofilm phenotype, except for ST235 isolates WH-SGI-V-07622 and WH-SGI-V-07625, as well as isolates WH-SGI-V-07498 and WH-SGI-V-07624. Nevertheless, this analysis suggests many higher and lower density biofilm formers are closely related, and that biofilm production on stainless-steel is not necessarily predictable based only on genomic analysis.

**Fig. 7:**
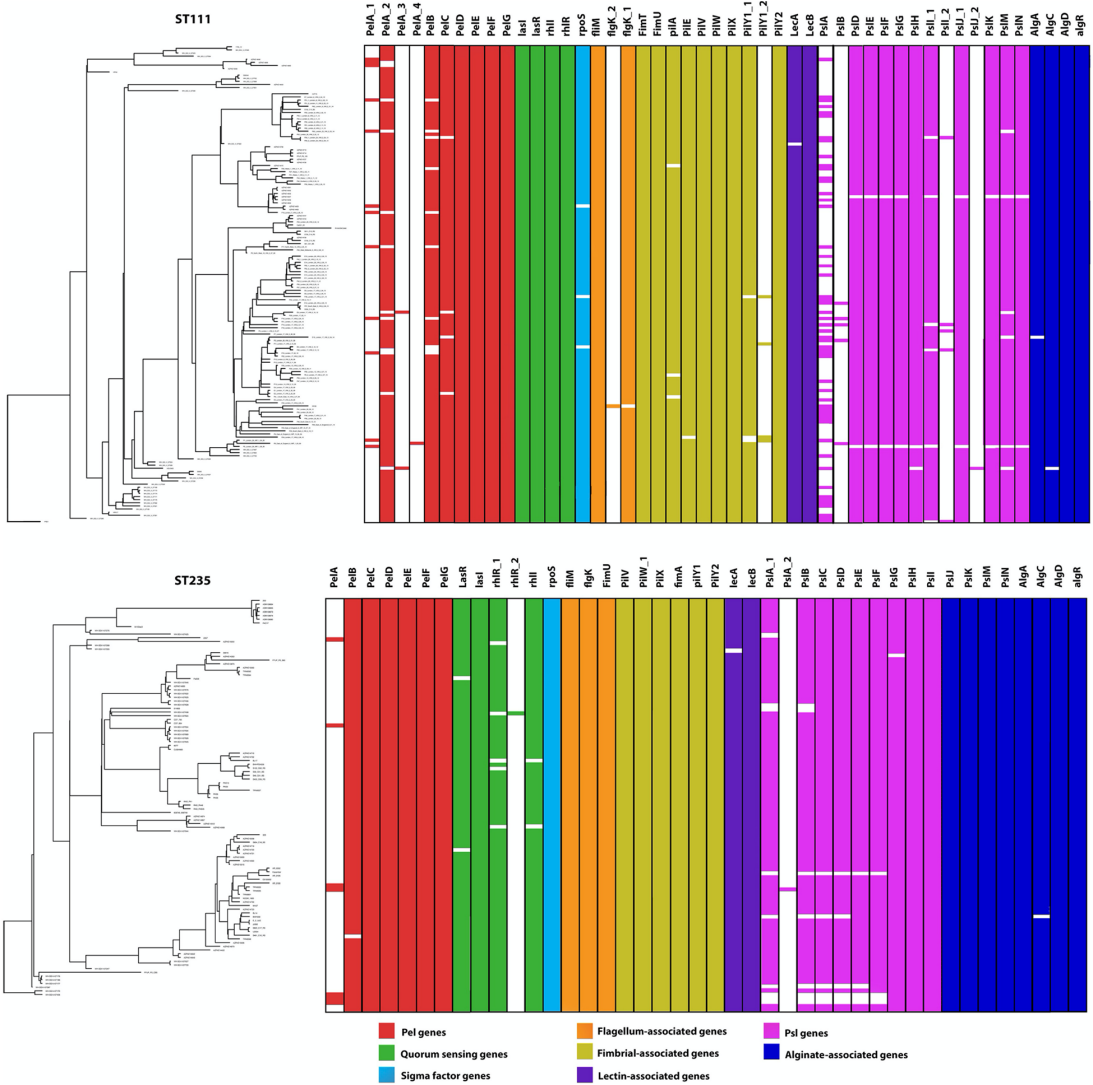
ST111 (top) and ST235 (bottom) biofilm gene presence/absence aligned with BEAST 2 analysis (Fig. 6). Coloured blocks represent 48 ST111 and 42 ST235 genes which have previously been linked to biofilm formation in the literature, whilst white space represents gene absence. Colour of each block dictates gene families or biofilm features described in figure legend

The pan-genome of biofilm genes widely described in the literature (Fig. 7) has a different core vs. accessory structure in each of the two lineages in this study, with the majority of genes (approximately 54 %) in both lineages, present in fewer than 15 % of genomes. The ST235 isolates analysed here have a higher percentage of core genes compared to ST111 (61.7 % vs. 31.34 %), whilst ST111 has a larger cloud genome compared to ST235 (40.3 % vs. 17.02 %). This variation is due to a greater number of homologues of genes in the ST111 biofilm pan-genome *pelA* has four homologues in ST111 isolates and *flgK*, *pilY1, pslI and pslJ* have two each. The genes *pilA* and *fimT* were both identified in the ST111 pan-genome, but not found in the ST235 pan-genome, whilst *pslC* was found in ST235 but not in the ST111 pan-genome.

Pan-genome association analysis of stainless-steel biofilm phenotype using Scoary on ST111 (*n* = 25) and ST235 (*n* = 23) isolates showed no significant associations after Bonferroni or Benjamini-Hocht corrections for multiple sampling (*p* < 0.05). However, Pyseer allows pangenome association analysis using mean OD_540_ values and the most statistically significant associations between accessory gene presence and biofilm production on stainless-steel are shown in Table 3. In ST111 and ST235 the most significantly associated genes are small proteins of 84 and 86 amino-acids respectively that share 21 % amino acid sequence identity to each other and only match to hypothetical proteins using blastp. HHpred indicate partial but significant structural similarity to the same *H. pylori* Type IV secretion system translocase for both proteins. Protein translocation by these previously uncharacterised putative translocases could have a significant role in biofilm production through extracellular transport contributing to biofilm mass. In ST111 isolates other genes with significant associations include the sensor kinase *cusS* (also associated with temperature dependent biofilm production - above) found in 22 isolates, and eight genes located together and present in only two ST111 isolates (isolate WH-SGI-V-07174 genes 02964–02971) that include two transposases, a type II toxin/antitoxin (TA) system RelE/ParE family toxin, and an XRE family transcriptional regulator that possibly represents an integrated plasmid sequence. Secreted TA system toxins have previously been found to be involved in biofilm formation in *P. aeruginosa* as well playing important roles in pathogenicity and persistence [44]. In ST235 genes associated with enhanced biofilm production on stainless-steel include two genes involved in mercury resistance, two methyltransferase proteins and an alginate biosynthesis gene *algA_3*. The putative ST235 T4SS translocase (WH-SGI-V-07406 gene 05831) is present in five ST235 isolates as are three genes in close proximity (05832, 05844 and 05845 in this isolate) that may represent part of a mobile genetic structure.

**Table 3:** Details of genes / COGs associated with biofilm phenoptype on stainless-steel identified by Pyseer

**ST111**					
**Cog / Gene**	**Function**		**Presence**	**lrt-*p*value**	**Beta**
group_257	Putative Type IV secretion system translocase	WH-SGI-V-07174_06314	16	0.005	-0.773
cusS_2	Sensor kinase CusS	WH-SGI-V-07168_02759	22	0.008	1.050
group_2942	HD-GYP domain-containing protein	WH-SGI-V-07174_02964	2	0.008	-1.820
group_2943	Hypothetical	WH-SGI-V-07174_02965	2	0.008	-1.820
group_2944	Hypothetical	WH-SGI-V-07174_02966	2	0.008	-1.820
group_2945	Transposase	WH-SGI-V-07174_02967	2	0.008	-1.820
group_2946	XRE family transcriptional regulator	WH-SGI-V-07174_02968	2	0.008	-1.820
group_2947	type II toxin-antitoxin system RelE/ParE family toxin	WH-SGI-V-07174_02969	2	0.008	-1.820
hin_4	DNA-invertase	WH-SGI-V-07174_02970	2	0.008	-1.820
group_2949	Tn3 family transposase	WH-SGI-V-07174_02971	2	0.008	-1.820
**ST235**					
**Cog / Gene**	**Function**		**Presence**	**lrt-*p*value**	**Beta**
group_2587	Putative Type IV secretion system translocase	WH-SGI-V-07406_05831	5	0.000	1.640
group_2588	Hypothetical	WH-SGI-V-07406_05832	5	0.000	1.640
group_2593	HK97 gp10 family phage protein	WH-SGI-V-07406_05844	5	0.000	1.640
group_977	Methyltransferase domain protein	WH-SGI-V-07406_05845	5	0.000	1.640
group_129	Glycoside hydrolase family 19 protein	WH-SGI-V-07425_03679	4	0.001	1.920
group_2655	Hypothetical	WH-SGI-V-07425_03618	4	0.001	1.920
merP_2	Mercuric transport protein periplasmic component	WH-SGI-V-07176_04878	6	0.001	-2.240
group_953	Mercury resistance co-regulator MerD	WH-SGI-V-07176_04876	6	0.001	-2.240
group_596	Phage gp6-like head-tail connector protein	WH-SGI-V-07406_05848	12	0.001	1.560
dcm	DNA-cytosine methyltransferase	WH-SGI-V-07170_03311	19	0.002	2.600
algA_3	Alginate biosynthesis protein AlgA	WH-SGI-V-07170_03301	19	0.002	2.600
group_199	SAM-dependent methyltransferase, partial	WH-SGI-V-07176_05804	4	0.003	2.420

Presence = number of genomes gene is present in /25 for ST111 and /23 for ST235

Core genome SNPs associated with biofilm production on stainless-steel were found using Pyseer and the most significant hits are shown in Table 4. In ST111 these include a SNP in a GGDEF and EAL domain-containing protein. Such proteins are known to have a key role in cell signalling and biofilm production [45]. The chemotaxis protein McpU and the PAS domain-containing protein are involved in cell-signalling and play a role in biofilm formation. Polymorphisms in the membrane proteins MprF and the DedA membrane family protein may have an effect on biofilm formation through increased translocation of proteins and other macromolecules from the cell. In ST235 the most significant core genome SNP is in a tRNA-Asn region however its relevance to biofilm production on stainless-steel is unclear. Four significant SNP sites are present in the topoisomerase primase domain-containing protein DnaG although the possible role of primase genes in biofilm production or regulation is obscure. Similarly, the importance of SNPs associated with biofilm phenotype for GTPase, ViaA and the two glycoside hydrolase family 19 proteins is unclear although we theorise that such glycoside hydrolases could be involved in biofilm matrix hydrolysis promoting transition of *P. aeruginosa* from a sessile to a planktonic state.

**Table 4:** Details of SNPs with significant associations with biofilm phenotype on stainless-steel within ST111 and ST235 genomes. Genes containing > 1 SNP site are in bold

**ST111**			
**variant**	**Gene product**	**lrt-*p*value**	**beta**
1420097_C_T	Transglycosylase SLT domain-containing protein	7.790E-03	1.160E+00
5765510_C_T	GGDEF and EAL domain-containing protein	7.790E-03	1.160E+00
3148782_G_A	hypothetical protein	7.790E-03	1.160E+00
5733260_G_A	Putative 2-dehydropantoate 2-reductase	7.790E-03	1.160E+00
3227055_G_T	DedA membrane protein family	7.790E-03	1.160E+00
460063_G_T	EcsC family protein	7.790E-03	1.160E+00
1590045_A_G	Methyl-accepting chemotaxis protein McpU	7.790E-03	1.160E+00
788564_G_A	Integral membrane protein mprF [Brucella suis]	7.790E-03	1.160E+00
124758C_G	PAS domain-containing protein	9.980E-03	9.950E-01
**ST235**			
**variant**	**Gene**	**lrt-*p*value**	**beta**
3107052_C_T	tRNA-Asn(gtt)	1.14E-05	1.95E+00
1737948_T_C	DUF1983 domain-containing protein	1.08E-04	2.18E+00
**1580887_A_G**	**GTPase**	**3.45E-04**	**2.33E+00**
1549594_C_T	Relaxase	3.45E-04	2.33E+00
**1580485_A_G**	**GTPase**	**3.45E-04**	**2.33E+00**
3128027_C_G	Glycoside hydrolase family 19 protein	3.52E-04	2.15E+00
4192259_C_G	Glycoside hydrolase family 19 protein (*n.b.* different gene to above)	3.52E-04	2.15E+00
1553088_A_G	Conjugal transfer protein TraG N-terminal domain-containing protein	4.58E-04	-2.91E+00
1565427_G_C	TIGR03758 family integrating conjugative element protein	4.58E-04	-2.91E+00
**3128516_G_A**	**Hypothetical protein**	**5.38E-04**	**1.68E+00**
**3128498_C_G**	**Hypothetical protein**	**5.38E-04**	**1.68E+00**
**312896T_G**	**VWA domain protein viaA**	**5.38E-04**	**1.68E+00**
1584342_T_C	DNA topoisomerase III	6.85E-04	1.82E+00
310354C_T	Type I restriction endonuclease subunit R	1.17E-03	2.64E+00
**3101715_A_T**	**topoisomerase primase domain-containing protein dnaG**	**1.17E-03**	**2.64E+00**
**3101859_T_C**	**topoisomerase primase domain-containing protein dnaG**	**1.17E-03**	**2.64E+00**
**3101802_G_A**	**topoisomerase primase domain-containing protein dnaG**	**1.17E-03**	**2.64E+00**
**3102546_C_T**	**topoisomerase primase domain-containing protein dnaG**	**1.17E-03**	**2.64E+00**
**312840T_C**	**VWA domain protein viaA**	**2.21E-03**	**1.55E+00**
309466T_G	Type I restriction endonuclease subunit R	2.92E-03	1.74E+00

## Discussion

In this study we demonstrate the ability of recently developed bacterial genome and pangenome analysis tools to discover accessory genes and core-genome SNPs that are associated with two different biofilm phenotypes. This resulted in our discovery of a genetic region containing many heavy metal resistance genes (arsenic, copper, mercury and cadmium) that we found associated with increased biofilm production at 22 °C compared to 37 °C and core genome SNPs in biofilm associated genes such as *algA*, *algX*, type II secretion system protein D gene *xpsD* and response regulator genes such as *pleD* and *quiP* that are also significantly associated with this phenotype and have previously been shown to have roles in biofilm biosynthesis and quorum sensing. Pangenome associations with biofilm production by isolates of ST111 and ST235 on stainless-steel similarly revealed novel accessory genes including ones with homology to a T4SS translocase and a type II TA system toxin protein as well as an alginate accessory gene *algA_3* and two mercury resistance genes. We also identified several SNPs in cell-signalling and cell-membrane protein genes that are associated with enhanced biofilm production on stainless-steel. It is not clear why possession of genetic regions coding for heavy metal resistance should results in enhanced biofilm production at lower temperatures but we hypothesize that such regions may be associated with overall genotypes that have adapted for survival in such environments.

Whilst the phenotype and genotype analysis and associations described in this paper provide novel insight into the relationship between non-discreet phenotypes and genotype, standardization of such phenotypes are required. This will be essential if GWAS and pan-GWAS studies are to be utilized to better understand the genetics of biofilm production. Central to this is the choice of methodologies for association analysis. In our pangenome analysis we found Scoary to be useful in identifying accessory genes associated with biofilm phenotype in our larger sample experiments (temperature-dependent biofilm) but lacked power in studying smaller groups of isolates. Pyseer proved more useful in analysing smaller datasets (stainless-steel biofilm analysis) in this study possibly due to the inclusion of continuous phenotype data rather than binary phenotypes in association studies that is not possible using Scoary. When both Scoary and Pyseer were used to analyse the same large dataset (280 isolates) they both identified many of the same accessory genes (Table 1) although for Scoary we found the use of Bonferroni (FWER) corrected *p*-values was too restrictive and Benjamini-Hochberg (FDR) corrected *p*-values may be more appropriate to such analyses.

## Conclusions

We were able to identify candidate genes in which polymorphism (or deletion) was associated with differences in temperature-dependent biofilm phenotype for 280 *P. aeruginosa* isolates. The two different GWAS approaches used in this paper (SNP-based and gene presence / absence) both resulted in the identification of some similar genes, The relationships between biofilm phenotypes on stainless-steel and genotype in *P. aeruginosa* was less clear, likely due to the smaller number of isolates used in this study. Further work should seek to utilize GWAS and pan-GWAS studies to identify new gene targets / cellular machinery alongside functional studies such as gene expression (for example RNA-seq). Our study demonstrates the importance of understanding the complex nature of *P. aeruginosa* biofilm, and that phenotypic studies in isolation and/or assumptions that biofilm phenotype is consistent across *P. aeruginosa* offer little in the way of meaningful data. Unravelling the complexity of phenotype-genotype relationships of *P. aeruginosa* will be an essential step in understanding and targeting *P. aeruginosa* biofilms as part of possible therapeutic strategies to tackle the threat posed by this major MDR pathogen.

## Methods

### Bacterial isolates and genome sequences

Isolates in this study were collected and genome sequenced in a previous study by van Belkum et al., 2015 [6]. They comprise isolates from the bioMérieux private clinical strain collection (218 isolates) and isolates from a study by Pirnay et al., 2009 [10] (62 isolates). This study analysed two different sets of these *P. aeruginosa* isolates. The first set, hereafter described as ‘temperature-dependent biofilm analysis’ contained all 280 isolates details of which are shown in Suppl. Tables 1, this study and Suppl. Table ST1 in van Belkum et al., 2015 [6].

The second set, hereafter described as ‘stainless-steel biofilm analysis’ contained 260 *P. aeruginosa* genomes (Suppl. Table 2), 153 of which belong to ST111 and 107 to ST235. Of these, 25 ST111 and 23 ST235 isolates were used to study biofilm phenotypes *in vitro*. All genomic data were downloaded from Genbank as Fasta nucleotide sequences. Bacterial isolates used for biofilm phenotyping were provided by bioMérieux (France) and Synthetic Genomics (USA) and were analysed in two previously published studies [6, 7].

### Construction of phylogenies

Core genome Single Nucleotide Polymorphisms (SNPs) were identified using the kSNP3 (v3.1) pipeline [26]. Kmer lengths of 21 were used for temperature-dependent biofilm analysis, whilst for stainless-steel biofilm analysis, kmer lengths for ST111 and ST235 were 21 and 23 nucleotides respectively. Kmer lengths were selected using the KCHOOSER module within the kSNP3 suite which provides the optimum kmer length for each dataset. Bayesian phylogenetic analysis was performed using BEAST 2 (v2.4.7) [46]. The following conditions were set based on those used in a phylogenetic study of the *P. aeruginosa* MDR ST111 lineage [9] - gamma site heterogeneity model, Hasegawa-Kishino-Yano (HKY) substitution model, relaxed-clock log-normal, chain length 5,000,000. BEAST 2 output was summarized using TreeAnnotator with a 5 % burn in.

### Construction and interrogation of pan-genome

All genomes were annotated using PROKKA (v1.12) [47]. Genome Feature Files (GFF3) produced by PROKKA were used as input for Roary (v3.11.2) [48] pan-genome analysis using default settings. In brief Roary; converts input files into protein sequences, filters partial sequences and then performs an all-against-all comparison with BLASTP with a default percentage sequence identity of 95 %. Outputs were visualized with the interactive visualization tool Phandango v1.3.0 [49]. Pangenome terminology is used as described by Roary; for example core genes (99 % <= strains < = 100 %), cloud genes (0 % <= strains < 15 %). Non-core genes are considered accessory genes whilst the pan-genome is considered to be all the genes found within a cohort of genomes.

### Biofilm phenotypes – Temperature-dependent biofilm analysis

The method for biofilm growth was a modified version of that described by Coffey and Anderson [50]. *P. aeruginosa* strains were maintained on Luria-Bertani (LB) agar (BD, Sparks, MD) at 4 °C. Liquid cultures were prepared by inoculating a single colony into 10 ml of LB broth (BD, Sparks, MD), and incubated overnight at 37 °C with agitation (180 rpm). This was diluted to an optical density (OD) of 1.0_540_ and a 1:100 inoculation in sterile LB broth was made in standard non-modified 96-well polystyrene plates (Starsted, Leicester, UK). Plates were incubated without shaking at either 22 or 37 °C for 48 h.

### Biofilm phenotypes – Stainless-steel biofilm analysis

Stainless-steel biofilm analysis was performed on MBEC biofilm 96-well plates (Innovotech, Edmonton, Canada), with pegged lids spray coated with stainless-steel. The sub-micron thick stainless-steel coatings were deposited onto the lid by the physical vapour deposition technique of magnetron sputtering, which is widely used in industry to deposit thin (sub-micron to several micron thick) metallic or ceramic coatings onto a wide range of components [51]. The coatings were sputtered from a 300mm x 100mm 304 grade stainless-steel target in a UDP350 coating rig (Teer Coatings Ltd).

An overnight culture of each strain was diluted to an optical density (OD) of 1.0_540_. A 1:100 inoculation in sterile LB broth was made in a well of the MBEC plate base (*n* = 4 per isolate).

The MBEC lid was replaced and the plate was incubated at 37 °C with agitation (110 rpm) for 48 h, with a change to a new 96-well plate with 200 µl of sterile LB broth in each well after 24 h.

### Biofilm phenotypes – quantifying

To quantify biofilms, plates or pegs were gently immersed in water to remove non-adhered cells, placed in 0.1 % crystal violet (w/v) (Sigma, Dorset) for 10 min then gently washed in water, twice, to remove excess stain. The plates for temperature-dependent biofilm analysis had 200 µl of 30 % acetic acid (v/v) added to each well to solubilize stain, whereas the lid with stained pegs was placed into a 96-well plate containing 200 µl per well of 30 % acetic acid (Fisher Scientific, Loughborough) for 10 min with agitation (110 rpm). Optical density of each well was measured at 540nm using a FLUOstar Omega plate reader (BMG Labtech, Aylesbury).

### Trait definition

In order to carry out GWAS and pan-GWAS (below), categorization of biofilm production was required. Currently, no agreed method exists for the categorization of higher density or lower density biofilm formers, and the wider biofilm literature uses a dynamic range of methods, characteristics and approaches to such studies [52]. For temperature-dependent biofilm analysis, isolates were split into one of two traits: either producing more biofilm at 37 °C compared to growth at 22 °C, or producing more biofilm at 22 °C compared to 37 °C (Fig. 2 and Suppl. Table 1). As part of our stainless-steel biofilm analyses, a ‘higher density’ biofilm formation trait was considered to be exhibited in strains providing an OD reading > = 0.5_540_, whilst ‘lower density’ biofilm formation was considered as an OD reading of < 0.5_540_. Defining the stainless-steel biofilm trait as such provides statistically significant differences (*p* < 0.05) when comparing the two sets of optical density data. Similar approaches to arbitrarily classifying continuous phenotypes have been used in previous bacterial GWAS studies [53].

### Pan-genome-wide association analysis

We used two GWAS analysis tools to investigate pangenome associations with biofilm phenotypes:- Scoary (v.1.6.9) [54] and Pyseer (v.1.3.3) [55]. These have been developed specifically for the study of bacterial datasets. The input for both Scoary and Pyseer are the gene presence / absence file generated by Roary and a traits file containing isolate name and either a binary or categorical value. Pyseer can also use quantitative (continuous) phenotype data as input. Associations in Scoary are derived from scoring the correlation of each gene in the accessory genome with phenotype using Fisher’s exact test before correcting for spurious associations caused by stratification due to phylogenetic structure. The key outputs of Scoary are a list of genes or Common Orthologous Groups (COGs) with an odds ratio and associated *p-*values corrected for erroneous false positives due to multiple testing using both false discovery rate (FDR) [56] and family-wise error rate (FWER) [57] approaches commonly called Benjamini-Hochberg and Bonferroni corrections respectively. Pyseer uses linear regression models adjusted for stratification in population structure and outputs a beta value (slope of the regression line) with associated likelihood ratio test (lrt) *p-*values.

Analyses were performed independently for temperature-dependent biofilm traits as for both ST111 lineage and ST235 lineage genomes in the stainless-steel biofilm analysis. Genes found to have statistically significant associations with biofilm phenotype were further annotated using Blastp and if no hit was found, assessed using the protein homology detection, function and structure prediction tool HHpred (MPI Bioinformatics Tool Kit) [58]. The top three HHpred predictions were considered for assumed protein identity and function, assuming a match probability of at least 80 %.

### SNP genome-wide association study

In the temperature-dependent biofilm analysis core genome SNPs relative to a PAO1 reference genome [59] were identified using kSNP3 and we then used Pyseer to identify SNPs associated with biofilm phenotype. Core genome SNPs were identified separately in our stainless-steel biofilm analyses by kSNP3 using ST111 strain FRD1 [60] and ST235 strain NCGM2 [61] as references. Analysis was performed independently for temperature-dependent analysis as before for both ST111 lineage and ST235 lineage genomes in the stainless-steel dataset. The stainless-steel dataset was analysed by Scoary using binary phenotype data (1 = OD_540_ > = 0.5 and 0 = OD_540_ < 0.5) and by Pyseer using continuous data (mean OD_540_ values). SNPs significantly associated with phenotype were located in the PAO1 genome using Artemis genome browser [62]. Genes containing SNPs of interest were interrogated using BlastP and HHpred, with the top three predictions with a probability match of over 80 % considered for assumed protein function.

## Supplementary Information

**Additional file 1:** Suppl. **Figure S1**. Optical density readings at 540nm of *Pseudomonas aeruginosa* biofilm after staining with 0.1 % crystal violet and solubilised in 30 % acetic acid. Biofilms were grown on a modified MBEC assay plate, of which the pegs had been coated in stainless-steel. Data represent twenty-five ST111 (A) and twenty-three ST235 (B) strains. An optical density cut off of 0.5_540_ was used to differentiate the statistically significant groups of high density (green) and low density (red) biofilm producers. Significance (*P* < 0.05), as assessed by T-Test is denoted by *. Error bars represent standard deviation. *n* = 4 for each isolate. Suppl. **Figure S2**. Optical density related to biofilm stained with 0.1 % crystal violet and solubilised in 30 % acetic acid at either 22 or 37 °C two independent *Pseudomonas aeruginosa* PAO1 transposon insertion *arsR* mutants compared to WT. Error bars represent standard deviation. *n* = 4 for each isolate/temperature condition. Details of the transposon mutants are available at:- http://www.pseudomonas.com/feature/show/?id=107334&view=transposons.

**Additional file 2:**

## Data Availability

Genbank accession numbers for all genome assembly sequences are shown in Suppl. Table 1. Phenotypic data for temperature-dependant biofilm analysis and stainless - steel biofilm analysis are shown in Suppl. Tables 1 and 2 respectively.

## Abbreviations

BLAST: Basic local alignment search tool
bp: Base pair(s)
COGs: Clusters of orthologous groups
GWAS: Genome-wide association study
ICE: Integrating conjugative element
kb: Kilobase pair(s)
lrt: Likelihood-ratio test
MDR: Multidrug resistant
MLST: Multilocus sequence typing
OD: Optical density
QS: Quorum sensing
SAM: S-adenosyl methionine
SNP: Single nucleotide polymorphism
ST: Sequence type
WGS: Whole genome sequencing
