# Supplementary figures and images for: Biofilm associated genotypes of multiple antibiotic resistant Pseudomonas aeruginosa
Source: BMC Genomics. 2021 Jul 26;22:572. doi: 10.1186/s12864-021-07818-5 (PMC8314537; doi:10.1186/s12864-021-07818-5)

Suppl. Figure 1

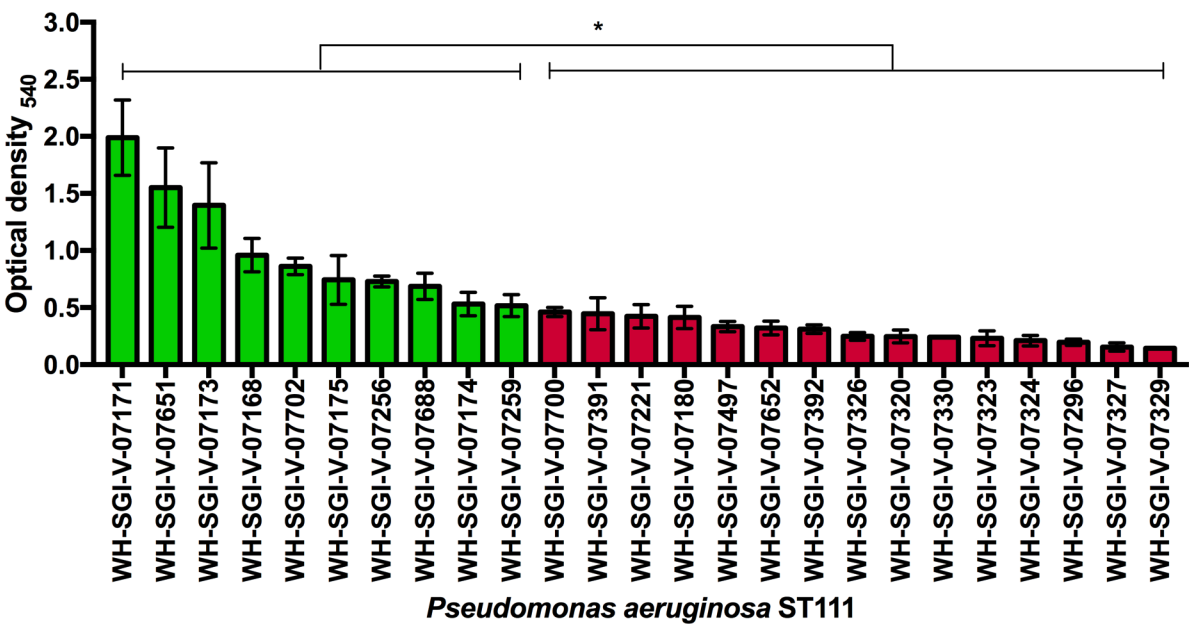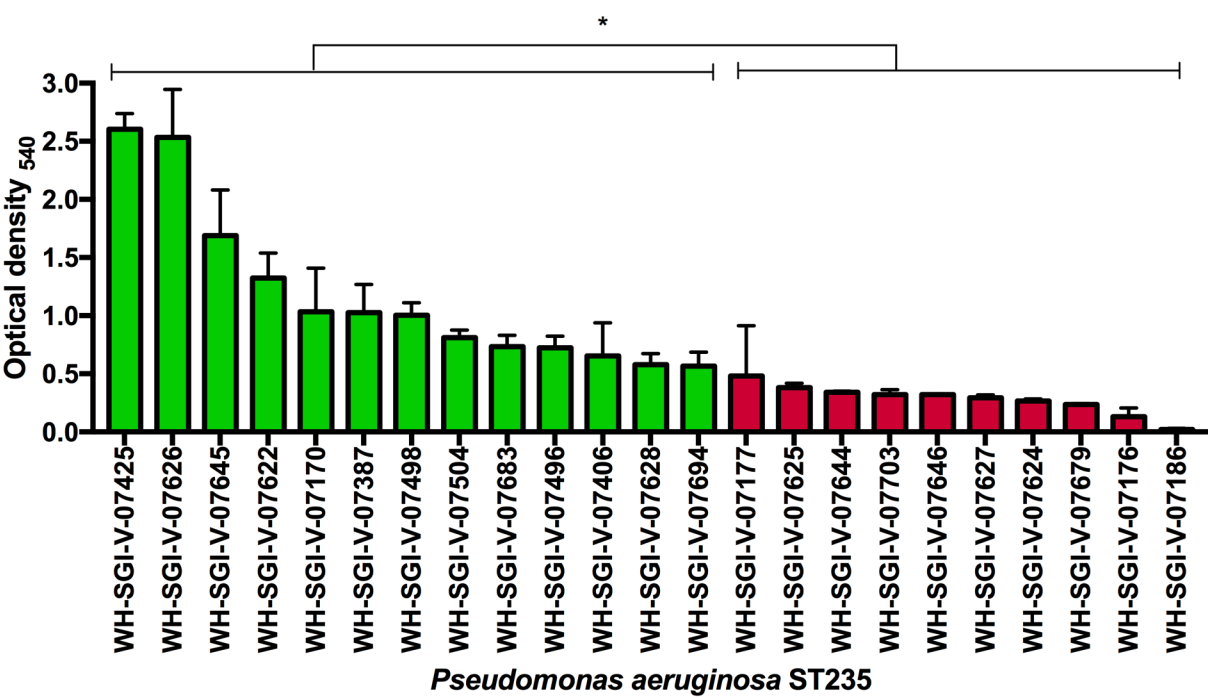

Suppl. Figure 2

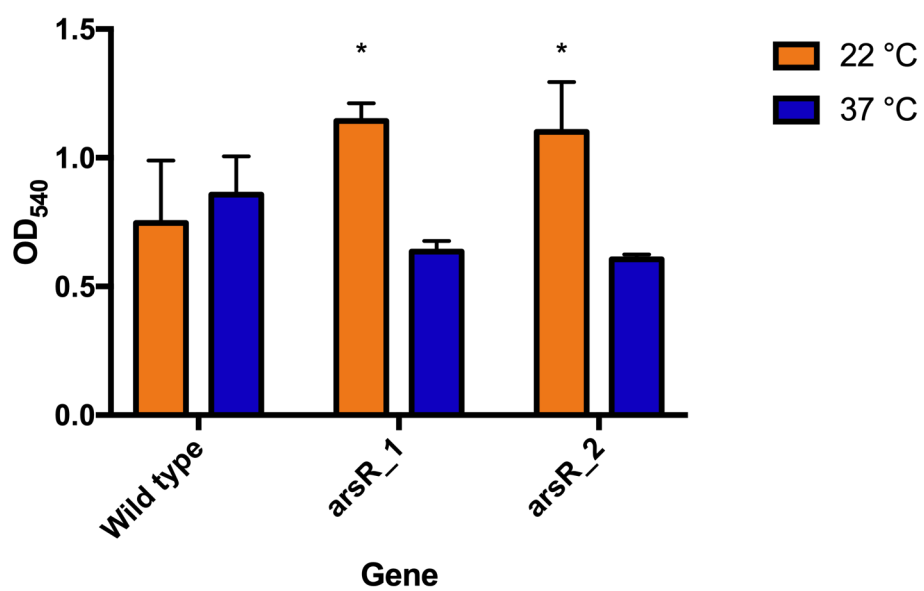

Supplement: Supplementary file 1 — Additional file 1: Suppl. Figure S1. Optical density readings at 540nm of Pseudomonas aeruginosa biofilm after staining with 0.1 % crystal violet and solubilised in 30 % acetic acid. Biofilms were grown on a modified MBEC assay plate, of which the pegs had been coated in stainless-steel. Data represent twenty-five ST111 (A) and twenty-three ST235 (B) strains. An optical density cut off of 0.5540 was used to differentiate the statistically significant groups of high density (green) and low density (red) biofilm producers. Significance (P < 0.05), as assessed by T-Test is denoted by *. Error bars represent standard deviation. n = 4 for each isolate. Suppl. Figure S2. Optical density related to biofilm stained with 0.1 % crystal violet and solubilised in 30 % acetic acid at either 22 or 37 °C two independent Pseudomonas aeruginosa PAO1 transposon insertion arsR mutants compared to WT. Error bars represent standard deviation. n = 4 for each isolate/temperature condition. Details of the transposon mutants are available at:- http://www.pseudomonas.com/feature/show/?id=107334&view=transposons. [file 12864_2021_7818_MOESM1_ESM.pdf]
